# Global and site-specific analysis of bone in a rat model of spinal cord injury-induced osteoporosis

**DOI:** 10.1016/j.bonr.2019.100233

**Published:** 2019-11-29

**Authors:** Jonathan A. Williams, James F.C. Windmill, K. Elizabeth Tanner, John S. Riddell, Sylvie Coupaud

**Affiliations:** a Department of Biomedical Engineering, Wolfson Building, University of Strathclyde, Glasgow G4 0NW, UK; b Department of Electronic and Electrical Engineering, Royal College Building, University of Strathclyde, Glasgow G1 1XW, UK; c Biomedical Engineering Division, James Watt School of Engineering, James Watt South Building University of Glasgow, Glasgow G12 8QQ, UK; d Institute of Neuroscience and Psychology, College of Medical, Veterinary and Life Sciences, University of Glasgow, G12 8QQ, UK

**Keywords:** Micro-computed tomography, Osteoporosis, Trabecular bone, Cortical bone, Spinal cord injury, Disuse

## Abstract

Micro-Computed Tomography bone analysis is the gold standard method for assessing trabecular and cortical bone microarchitecture in small animal bones. This technique reports morphometric parameters as averages over selected volumes of interest (VOIs). This study proposes the introduction of an additional global 2D morphometric step into the analysis process, that provides a survey of the underlying morphometric variation present throughout both trabecular and cortical bone. The visualisation of these morphometric distributions provides a systematic approach to VOI selection that provides rationale and adds confidence to subsequent 3D morphometric analysis. To test the applicability and value of this methodological addition it was applied to the distal femur of a rat model of spinal cord injury (SCI)-induced osteoporosis. The 2D morphometric variation of both trabecular and cortical bone was quantified as a function of bone length. SCI-induced osteoporosis was localised in i) trabecular bone, where metaphyseal bone was more severely affected than epiphyseal bone, and there was a significant reduction in Distal Femoral Trabecular Extent, a new parameter defined here that quantifies how far trabecular bone penetrates in to the marrow cavity, ii) cortical bone, where diaphyseal bone underwent significant lowering of both cortical area and thickness, while distal-metaphyseal bone did not. Theses site-specific changes were validated, further elucidated and compared with follow-up conventional 3D analysis. The techniques applied here are equally applicable to other long bones (tibia, humerus, radius, ulna), other types of imaging modality and other types of experimental design including the effects of rehabilitation, aging, loading, gene knockout and pharmacological intervention.

## Highlights

• 2D morphological surveying identifies regions warranting further 3D investigation.
• Trabecular microarchitecture site-specifically varies in the distal femur.
• SCI-induced osteoporosis changes metaphyseal more than epiphyseal trabecular bone.
• SCI-induced osteoporosis reduced the extent of metaphyseal trabecular bone.

## Graphical abstract

**Figure:**
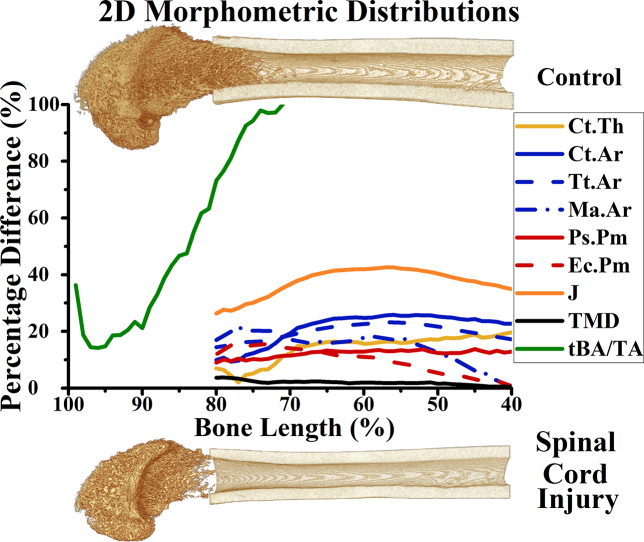

## 1 Introduction

Micro-Computed Tomography (μCT) is the gold standard technique for the assessment of trabecular and cortical bone micro-architecture, in the bones of mice and rats (Bouxsein et al., 2010). This pipeline most commonly consists of 6 main steps: scanning, reconstruction, orientation, segmentation, volume of interest (VOI) selection and 3D morphometric analysis. The end goal of these steps is to obtain representative morphometric parameters from 3D surface-rendered volumetric models of trabecular or cortical bone VOIs.

In this process the VOI selection step limits the scope of analysis to a relatively large sub-region of the bone. The architecture of bone can vary considerably over short distances, it is therefore imperative that every effort is made to identify appropriate VOIs for the relevant research question(s). The location and size of the VOIs are essential considerations. The location of all the VOIs must be such that anatomically and biomechanically similar VOIs are compared. While if the size of the VOI is not appropriate it may dilute site-specific effects, for example if a trabecular VOI is very long (e.g. extending far into the diaphysis), then the volume fraction of trabecular bone relative to a shorter VOI will be lower, conversely problems also occur when the VOI chosen is too small. 3D analysis performed on VOIs provides morphometric parameters as averages, therefore to acquire meaningful parameters, the variation of the parameter must be small enough such that substantial changes do not occur over dimensions which are of the same order of magnitude as the size of the VOI (Harrigan et al., 1988).

This study proposes the addition of a global 2D morphometric analysis step into the μCT analysis process. This step, which fits in between segmentation and VOI selection provides a more systematic approach to VOI selection compared to the commonly used set distance from an anatomical landmark method. Furthermore, it enables the acquisition of additional information that complements standard 3D analysis.

This step is global in that it does not require the selection of individual trabecular/cortical bone VOIs, the only limitation is the amount of bone scanned. This is important because conclusions from single sites do not generalise well to the whole bone and do not allow the quantification of site-specific effects. This step is 2D in that it is a slice-by-slice approach to morphometric analysis, except the final averaging step is omitted, allowing morphometric parameters to be expressed as a function of bone length, thus maintaining the spatial variability, directionality and extent of parameter distributions.

The primary aim of this study was to develop, apply, incorporate and demonstrate the value of a global 2D morphometric analysis step that complements the gold standard μCT analysis process. This step was applied to a model of disuse osteoporosis. Disuse osteoporosis describes a form of bone loss resulting from mechanical unloading of the musculoskeletal system (Alexandre and Vico, 2011). An extreme manifestation of disuse osteoporosis results from paralysis of the lower limbs after a complete spinal cord injury (SCI), leading to an increased fracture risk within this patient population (Lazo et al., 2001). Bone loss below the level of injury is in response to muscular paralysis as well as other factors including the spinal cord lesion and hormonal changes after injury (Jiang et al., 2006a). This response varies according to location along the bone (Rittweger et al., 2010), and type of bone (trabecular near the joints, cortical in the shaft) (Dudley-Javoroski and Shields, 2012). In patients with SCI, bone loss is generally most severe around the knee (distal femur, proximal tibia) (Biering-Sørensen et al., 1990; Dauty et al., 2000; Jiang et al., 2006b).

## 2 Materials and methods

### 2.1 Animals

Right femora were obtained from a rat model of SCI. The SCI model used was complete transection of the spinal cord at a low thoracic level (T9). 16 male Wistar rats were acquired from Harlan Laboratories, Loughborough, UK. Rats were 200–250 g (approximately 10–12 weeks old), equivalent to human adolescence (Sengupta, 2011). Rats were housed in pairs under a 12-h light/dark cycle with ad libitum access to food and water. All experimental procedures were approved by the Ethical Review Panel of the University of Glasgow and carried out in accordance with the Animals (Scientific Procedures) Act 1986.

### 2.2 Surgery and postoperative care

For surgery, rats were anesthetised with isoflurane and a laminectomy performed to expose the spinal cord at the T9 segmental level. In Control rats (*n* = 8) the wound was immediately closed. The SCI rats (n = 8) underwent spinal cord transections, using a method similar to that described by Lu et al. (2014). A small opening was made in the dura and the spinal cord cut transversely, at two locations, separated by approximately 1 mm, using iridectomy scissors (FST, No. 15002-08). A blunt 23G needle, connected to an aspirator, was used to remove a small amount of spinal cord tissue and any accumulating fluid. The completeness of the transection was confirmed visually by observing complete separation of the proximal and distal stumps through an operating microscope. The wound was closed, and rats recovered in warmed cabinets (28 °C) for up to 3 days, until thermoregulation was restored. Rats received analgesia (buprenorphine, 0.05 mg/kg and carprofen, 5 mg/kg subcutaneously at induction of anaesthesia and the morning after surgery). Saline (3–5 ml) and enrofloxacin (5 mg/kg) were given subcutaneously for 3- and 7-days post-surgery, respectively. The bladders of SCI rats were manually expressed three times a day until reflexive emptying returned (typically 12 to 14 day after surgery).

### 2.3 Preparation of bones

Rats were killed by anaesthetic overdose (Euthatal, Merial Animal Health Ltd., Harlow, UK) at 10 weeks after surgery. The femurs were dissected, all soft tissue removed, weighed (wet mass) and length (femoral length) measured. Femoral length was measured parallel to the femoral shaft, between the femoral head and distal condyles using digital callipers. The femurs were then wrapped in PBS-soaked gauze and stored at −20 °C.

No significant difference was observed in femoral length (Control 36.81 ± 0.25 mm, SCI 36.65 ± 0.28 mm), but wet masses were 19% lower in SCI than Control rats (Control 0.83 ± 0.02 g, SCI 0.67 ± 0.01 g, *p* < .0001).

### 2.4 μCT scanning protocols

**Fig. 1:**
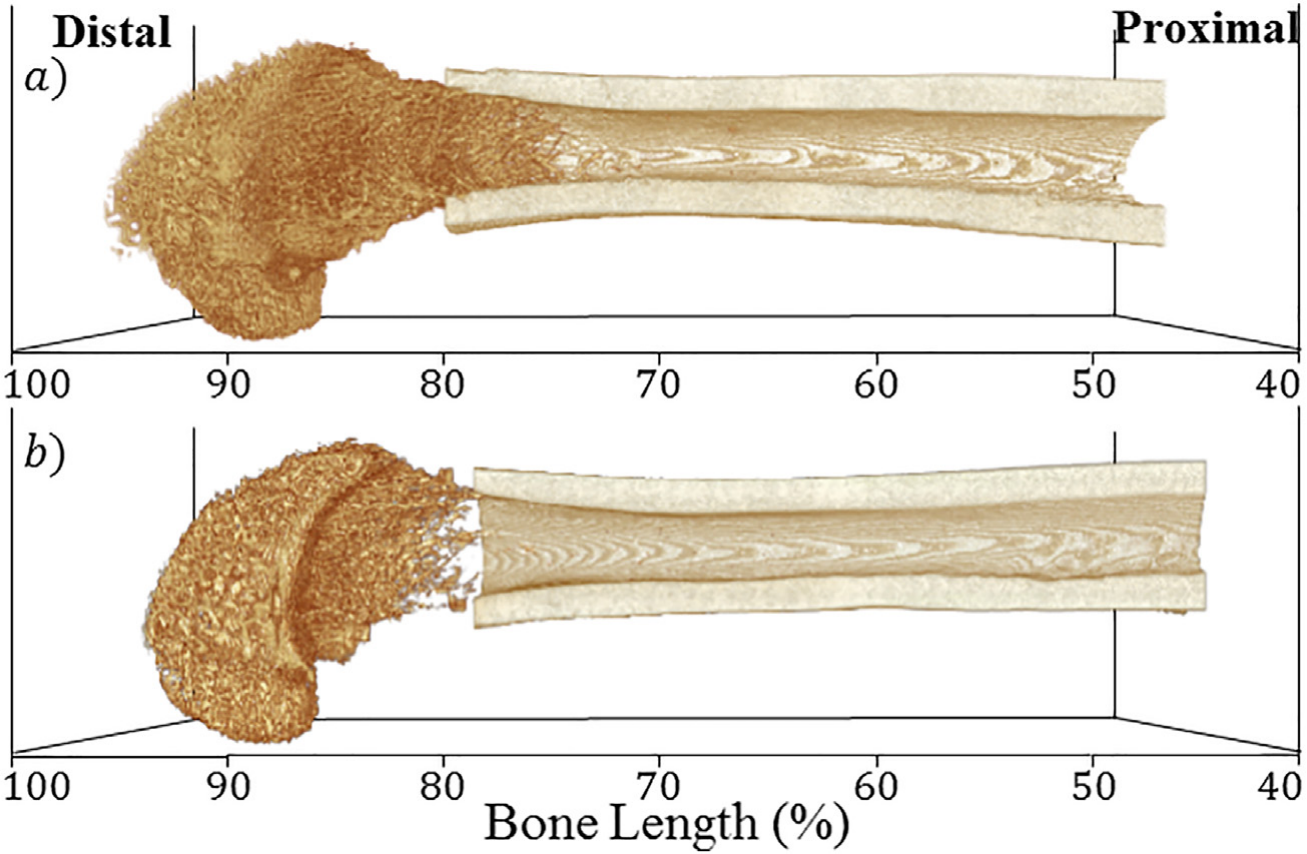
Representative μCT datasets of A) Control and B) SCI group 10 week-post surgery rat femurs. Segmented trabecular VOI spans 60 to 100% bone length and segmented cortical VOI spans 40 to 80% bone length.

The most distal 60% of the femur was scanned using a Bruker SkyScan 1172 μCT scanner (Kontich, Belgium), proceeding from the medial and lateral condyles past the epiphysis and growth plate into the metaphysis and cortical shaft (Fig. 1). All scans were performed in oversize mode using the following settings: 70 kVp X-ray tube voltage, 100 μA X-ray tube current, 470 ms integration time, 6.89 μm voxel size with 2 k camera resolution and 0.4° rotation step for a total of 180° with a 0.5 mm aluminium filter to reduce beam hardening artefacts. Projection images were reconstructed using SkyScan NRecon software (Version 1.6.9.18, Kontich, Belgium) into 8-bit grey level cross-sectional images, with the following reconstruction parameters: ring-artifact correction = 13, beam hardening correction = 40%, smoothing = 2.

### 2.5 2D morphometric analysis

The process used to obtain distributions that quantify the variation of trabecular and cortical 2D μCT morphometric parameters as a function of femoral length has three major steps.

#### 2.5.1 Image co-registration

A representative μCT scan was selected from each of the Control and SCI groups and used as a reference dataset. This reference scan was manually aligned to achieve a vertical femoral shaft centreline, such that the long axis of the femoral shaft was the z-axis of the dataset and that individual cross-sections (the x-y plane) were perpendicular to the femoral shaft, using DataViewer (Version 1.5.1.9, Kontich, Belgium). Thereafter all remaining μCT scans in each group (*n* = 7) were rigidly co-registered to this reference, in a semi-automated fashion again using DataViewer. This process is explained in full in the Supplemental Material, Section 1.

#### 2.5.2 Segmentation

Segmentation of trabecular bone from cortical bone was conducted automatically by creating a macro in CTAn (Version 1.14.10.0, Kontich, Belgium). The process is explained in full in the Supplemental Material, Section 2.

#### 2.5.3 Extraction of distribution

MATLAB scripts were written to extract 2D μCT morphometric parameter distributions from the co-registered binarised trabecular and cortical bone datasets (MATLAB 2015b, The MathWorks, Inc., Natick, Massachusetts, US). Scripts are available on request.

The trabecular morphometric parameters extracted were trabecular bone area fraction (BA/TA), trabecular thickness (Tb.Th_2D_), trabecular number (Tb.N_2D_), trabecular separation (Tb.Sp_2D_) and a new parameter termed trabecular extent (Tb.E). Tb.E quantifies the “vertical” distance in either percentage bone length- or millimetre-terms (mm used here), that the distal femoral trabecular structure extends into the medullary cavity from the distal growth plate reference level. It is quantified as a function of BA/TA, taken directly from the BA/TA distribution. The absolute TB.E (Tb.E_Abs_) quantifies the overall extent of distal trabecular bone within the 60 to 100% bone length segmented trabecular VOI. It is the distance between the growth plate reference level and the most proximal point on the distribution were BA/TA was greater than zero. All other Tb.E measures are the proximal distances from the distal femoral growth plate reference level to the first occurrence in the BA/TA distributions where BA/TA is equal to 30%, 20%, 10% and 0. These measures of Tb.E should not be considered a standard, they should be changed based on the trabecular structures of interest, for example, if BA/TA does not reach 30% in the SCI group, due to extensive changes after SCI, then there would be no point of comparison between Control and SCI group. The abbreviation of the distal femoral trabecular extent at BA/TA = 30% is either Tb.E_BA/TA=30%_ or Tb.E_30_. The distal femoral growth plate reference level was defined as the most proximal μCT slice in which there was a continuous chondrocyte seam. The cortical morphometric parameters extracted were cortical thickness (Ct.Th_2D_), cortical bone area (Ct.Ar_2D_), total area (Tt.Ar_2D_), marrow area (Ma.Ar_2D_), cortical area fraction (Ct.Ar_2D_/Tt.Ar_2D_), periosteal perimeter (Ps.Pm_2D_), endocortical perimeter (Ec.Pm_2D_) and second polar moment of area (J_2D_). Detailed summaries of each 2D morphometric parameter are available (Supplemental Material, Section 3).

For each slice in the co-registered, binarised datasets all 2D morphometric parameters were calculated and plotted as a function of bone length, such that they become morphometric distributions. Each femur within and between groups was of a different geometry and length, but the resolution used to scan them was identical, so each distribution was made up of a different number of μCT slices. To compare fixed points along the bone length (e.g. at 80% of total bone length) a cubic interpolation was performed on each distribution. The 2D morphometric parameters determined here were validated against 2D morphometric analysis performed using CTAn software (Version 1.14.10.0, Kontich, Belgium). See Supplemental Material, Section 4.

### 2.6 3D morphometric analysis

3D trabecular and cortical bone morphology was performed on sub-volumes of the same co-registered, binarised datasets to validate 2D findings and to provide additional microstructural information. VOI selection was guided by the 2D morphometric distributions. Two trabecular bone VOIs were selected. Firstly, a metaphyseal secondary spongiosa VOI spanning 81 to 85% bone lengthwas chosen as it contains the region of secondary spongiosa with the largest differences in BA/TA between Control and SCI. The more distal regions (85 to 89% bone length) are closer to the growth plate and thus contain more predominantly modelling primary spongiosa, and so were excluded from this particular analysis. More proximal regions (60 to 80% bone length) are nearer the terminal end of the distal femoral trabecular structure, where most of the trabeculae are contiguous with the cortex (Supplemental Material, Section 5), containing low volume fractions of trabeculae; thus, these were also avoided. Secondly, an epiphyseal VOI spanning 93 to 97% bone length was chosen as it was sufficiently away from the growth plate. Two cortical bone VOIs were also selected. Firstly, a diaphyseal VOI spanning 58 to 62% bone length was chosen to reduce the effects of the third trochanter, a bony projection prominent at 50% bone length, which is of minor importance in humans but is of noticeable size in rats. The third trochanter is indicated by the proximally increasing Ct.Th_2D_ and Ct.Ar_2D_ from approximately 40 to 55% bone length (Fig. 5). Secondly, a metaphyseal VOI spanning 81 to 85% bone length was chosen as it contains the region that is most reliably segmentable between samples and is not confounded by the effects of the growth plate and epiphyseal cortical shell, while also corresponding with the metaphyseal trabecular VOI. The standard trabecular morphometric parameters used were trabecular bone volume fraction (BV/TV), trabecular thickness (Tb.Th), trabecular number (Tb.N), trabecular separation (Tb.Sp), trabecular bone pattern factor (Tb.Pf), connectivity density (Conn.D), the bone surface area to volume ratio (BS/BV) and the un-plate index (uPi). uPi is the ratio of a structure's direct trabecular thickness (Tb.Th) to the thickness derived assuming a 2D plate-based model, it indicates the departure from an ideal plate morphology (Salmon, 2020). The cortical morphometric parameters used were cortical thickness (Ct.Th), cortical area (Ct.Ar), total area (Tt.Ar), marrow area (Ma.Ar), cortical area fraction (Ct.Ar/Tt.Ar), second polar moment of area (J) and eccentricity (Ecc) of the periosteum, determined as the ratio of the semimajor and semiminor axes, as calculated using CTAn software (Version 1.14.10.0).

### 2.7 Statistical analysis

Anderson-Darling and Kolmogorov-Smirnov tests were used to test for normality between the SCI and Control groups for all 2D and 3D morphometric parameters. No significant deviations from normal distribution (*p* < .05) were detected for any parameter reported. Student's *t*-test for independent samples was therefore performed for all 2D parameters at each 1% bone length, and for 3D parameters. All analysis was performed using MATLAB 2015b.

## 3 Results

### 3.1 The variation of trabecular bone as a function of bone length

**Fig. 2:**
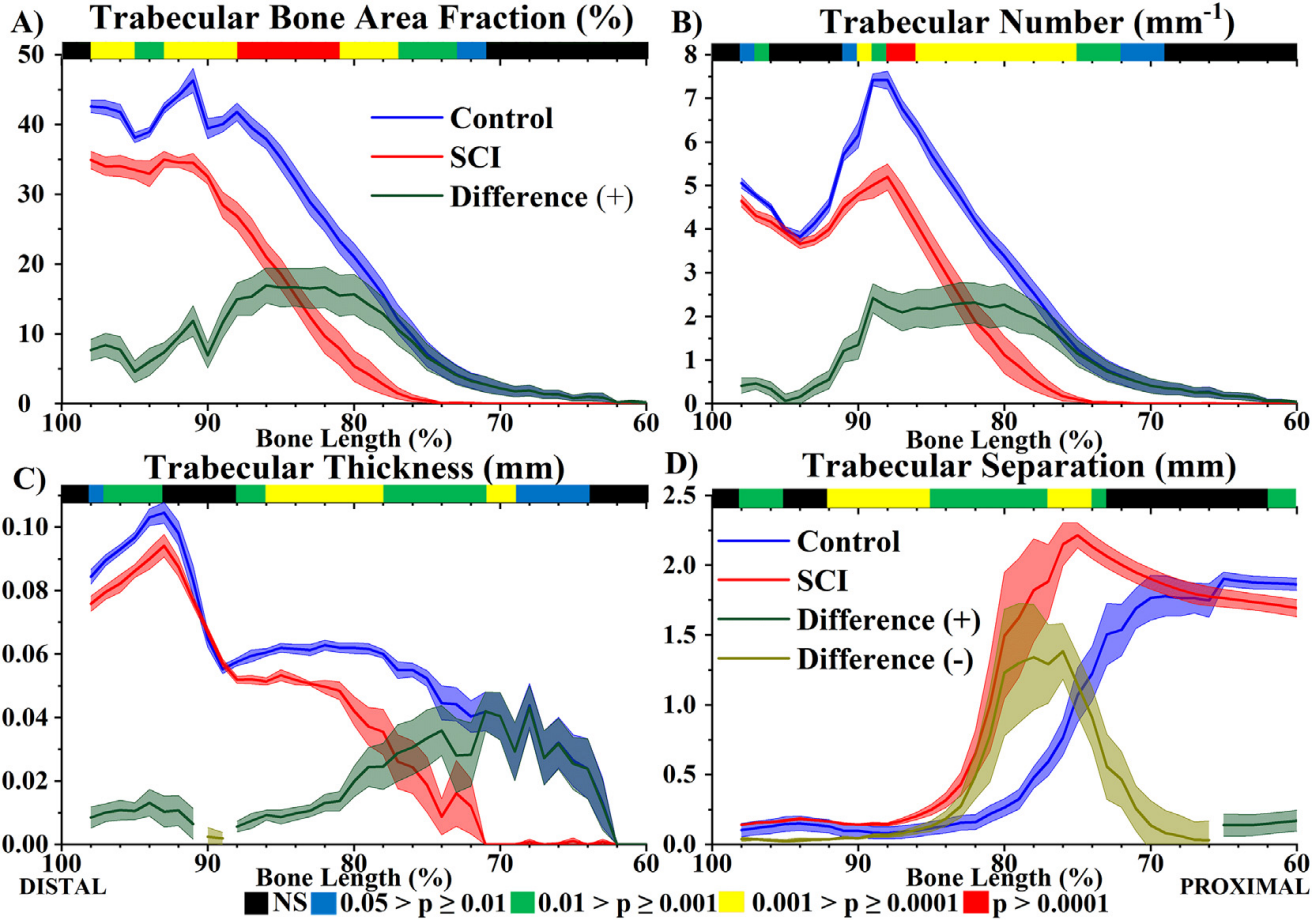
2D morphometric analysis of trabecular bone (60 to 100% bone length) at 10 weeks post-surgery, Control in blue, SCI in red (both *n* = 8). The absolute difference distribution (|Control – SCI|) is in green for positive and dark yellow for negative differences. A) Trabecular bone area fraction (BA/TA), B) Trabecular Number (Tb.N_2D_), C) Trabecular Thickness (Tb.N_2D_) and D) Trabecular Separation (Tb.Sp_2D_). Data shown as mean ± SE. Significant levels provided by *p*-value heat maps, where black is non-significant (NS), blue .05 > *p* ≥ .01, green .01 > *p* ≥ .001, yellow .001 > *p* ≥ .0001, red *p* > .0001. (For interpretation of the references to colour in this figure legend, the reader is referred to the web version of this article.)

Firstly, trabecular bone morphometric parameter distributions were analysed for the Control group, between 60 and 100% bone length from the proximal end of the femur (Fig. 2). Limited variation in epiphyseal BA/TA (91 to 98% bone length) was observed. In contrast, throughout the metaphyseal region (60 to 90% bone length) there was a rarefication of trabecular bone indicated by the monotonically decreasing BA/TA gradient, which ran proximally from the growth plate (at approximately 91% bone length) where BA/TA was highest, extending throughout the metaphysis and into the cortical shaft. Despite the limited variation in BA/TA throughout the epiphyses, Tb.N_2D_ and Tb.Th_2D_ varied significantly throughout (Fig. 2B & C). The maximum differences of Tb.N_2D_ and Tb.Th_2D_ within this compartment were 33% and 31%, respectively (both *p* < .001). Despite these microstructural changes Tb.Sp_2D_ remained approximately constant throughout (Fig. 2D). For metaphyseal trabecular bone, the Tb.N_2D_ distributions decreased monotonically with distance from the growth plate in a similar manner to BA/TA. In contrast, between 80 and 88% bone length Tb.Th_2D_ did not change significantly, such that the rarefication of trabecular BA/TA here was associated with a reduced number of trabeculae, but not reduced thickness, which resulted in a gradual increase in separation. More proximally (60 to 80% bone length), the decline in BA/TA was a combination of decreasing number and thickness, which over a very short distance resulted in a rapid increase in separation, to the point where Tb.Sp_2D_ represented the average diameter of the marrow cavity.

### 3.2 Site-specific trabecular bone changes as a result of SCI-induced osteoporosis

The effects SCI-induced osteoporosis had on the trabecular bone morphometric parameter distributions were also characterised (Fig. 2). At 10 weeks post-surgery, the distal femur of the SCI group exhibited site-specific patterns of trabecular bone changes. Compared to Control there was wide-spread significantly lower BA/TA (*p* < .05). A peak difference in BA/TA between SCI and Control of 17% (*p* < .0001) was observed at 86% bone length. The metaphyseal trabecular changes were characterised by lower Tb.N_2D_ and Tb.Th_2D_ throughout (both *p* < .05), which resulted in consistently higher Tb.Sp_2D_ (*p* < .01). For epiphyseal trabecular bone a less severe lowering of BA/TA (p < .01) was observed throughout, characterised by lower Tb.Th_2D_ (p < .05) between 93 and 98% bone length, lower Tb.N_2D_ (p < .05) only between 97 and 98% bone length and higher Tb.Sp_2D_ (p < .01) between 96 and 98% bone length.

**Fig. 3:**
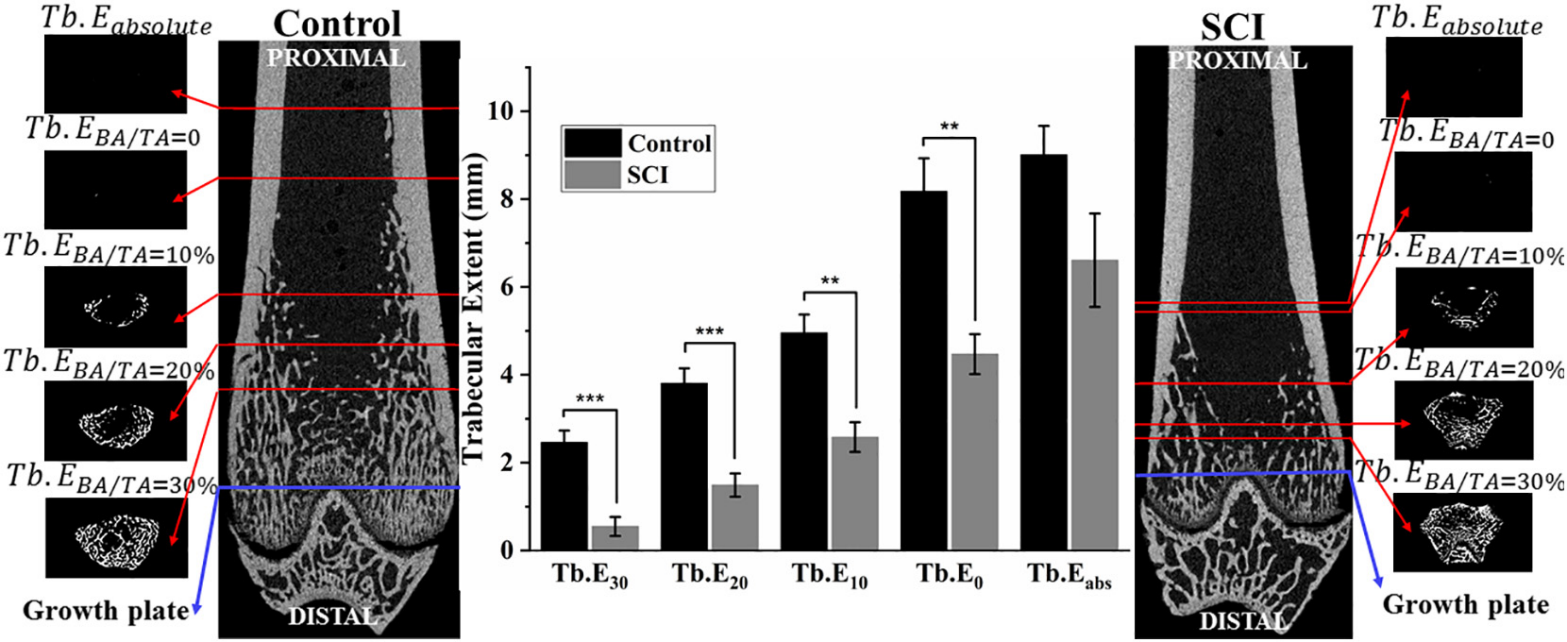
Trabecular extent (Tb.E) measured from the distal growth plate, proximal into the diaphysis, as a function of trabecular area fraction (BA/TA), at BA/TA = 30%, 20%, 10%, 0 (first occurrence) and absolute 0 (most proximal Tb.E) at 10 weeks post-surgery. Data shown as mean ± SE. ** and *** indicate *p* < .01 and *p* < .001 respectively. Coronal μCT slices for representative Control and SCI, are depicted with binarised cross-sectional slices at each Tb.E value quantified, depicting the BA/TA present at that slice.

The distal femoral trabecular extent (Tb.E), which quantifies how far trabecular bone penetrates into the medullary cavity from the distal growth plate, was significantly different at all BA/TA points of measure (Fig. 3). At 10 weeks post-surgery Tb.E at trabecular BA/TA equal to 30% (Tb.E_BA/TA=30%_) and Tb.E_BA/TA=0_ were 78% (*p* ≤ .001) and 45% (*p* ≤ .01) lower in SCI compared to the Control group, respectively.

**Fig. 4:**
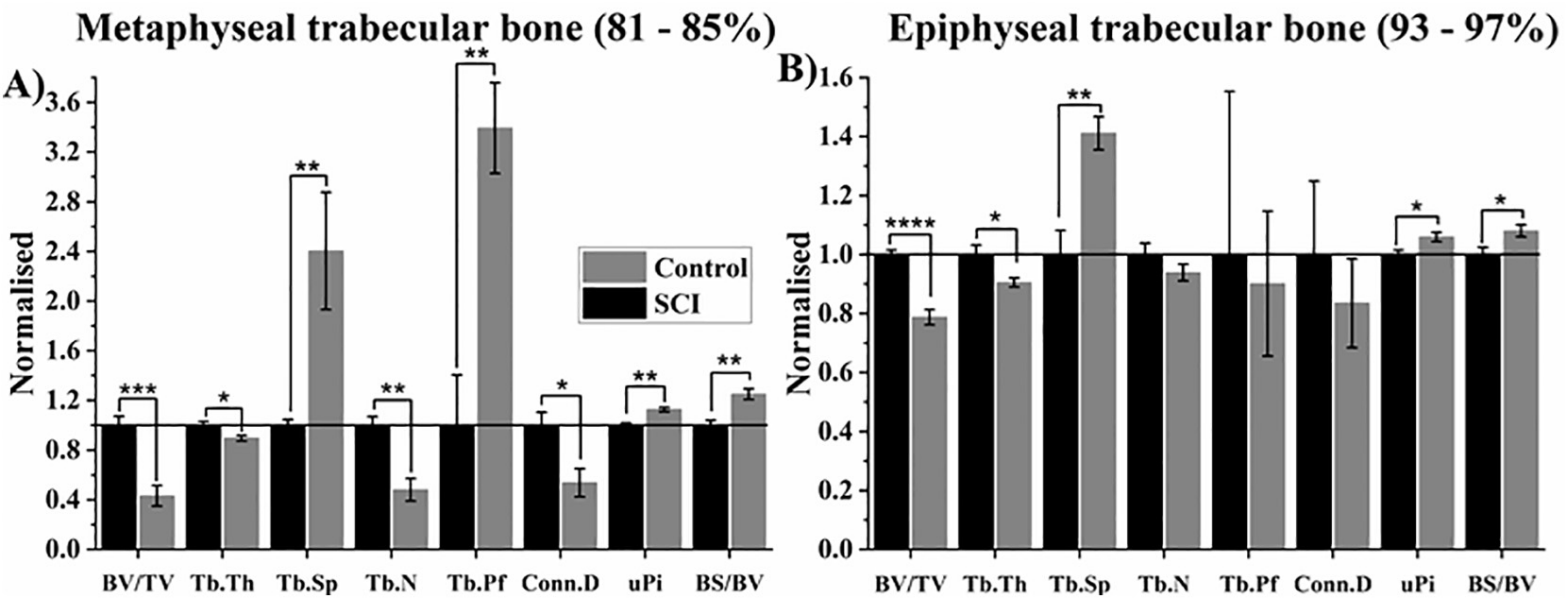
Average 3D morphometric parameters for A) metaphyseal and B) epiphyseal trabecular bone. All parameters are normalised with respect to the Control group. BV/TV: trabecular bone volume fraction; Tb: Trabecular; N: Number; Th: Thickness; Sp: Separation; Pf: Pattern Factor; Conn.D: connectivity density; uPi: un-plate index; BS/BV: bone surface area to volume ratio. Normalised average ± SE are presented. *, **, *** and **** indicate p < .05, p < .01, *p* < .001 and *p* < .0001, respectively.

These findings were validated with conventional 3D analysis (Fig. 4). The region identified to represent metaphyseal trabecular bone ranged from 81 to 85% bone length, selected because it contained secondary spongiosa with the largest differences in BA/TA between groups (Fig. 4). Other standard measures of trabecular bone morphometry were also assessed. For metaphyseal trabecular bone BV/TV was 57% (*p* < .001) lower compared to Control, characterised by 10% (*p* < .05) and 52% (*p* < .01) lower Tb.Th and Tb.N, respectively, with 140% higher average Tb.Sp (p < .01). These structural changes led to a 2.4-fold higher Tb.Pf (p < .01), 46% lower Conn.D (p < .05) and 13% and 25% higher uPi and BS/BV respectively (both p < .01) in SCI compared to Control. There was a less extreme 21% (*p* < .0001) lowering of epiphyseal BV/TV, characterised by 8% lower Tb.Th (*p* < .05) and 41% higher Tb.Sp (*p* < .01), leading to 6% and 8% higher uPi and BS/BV, respectively (both p < .05).

### 3.3 Variation of cortical bone as a function of bone length

**Fig. 5:**
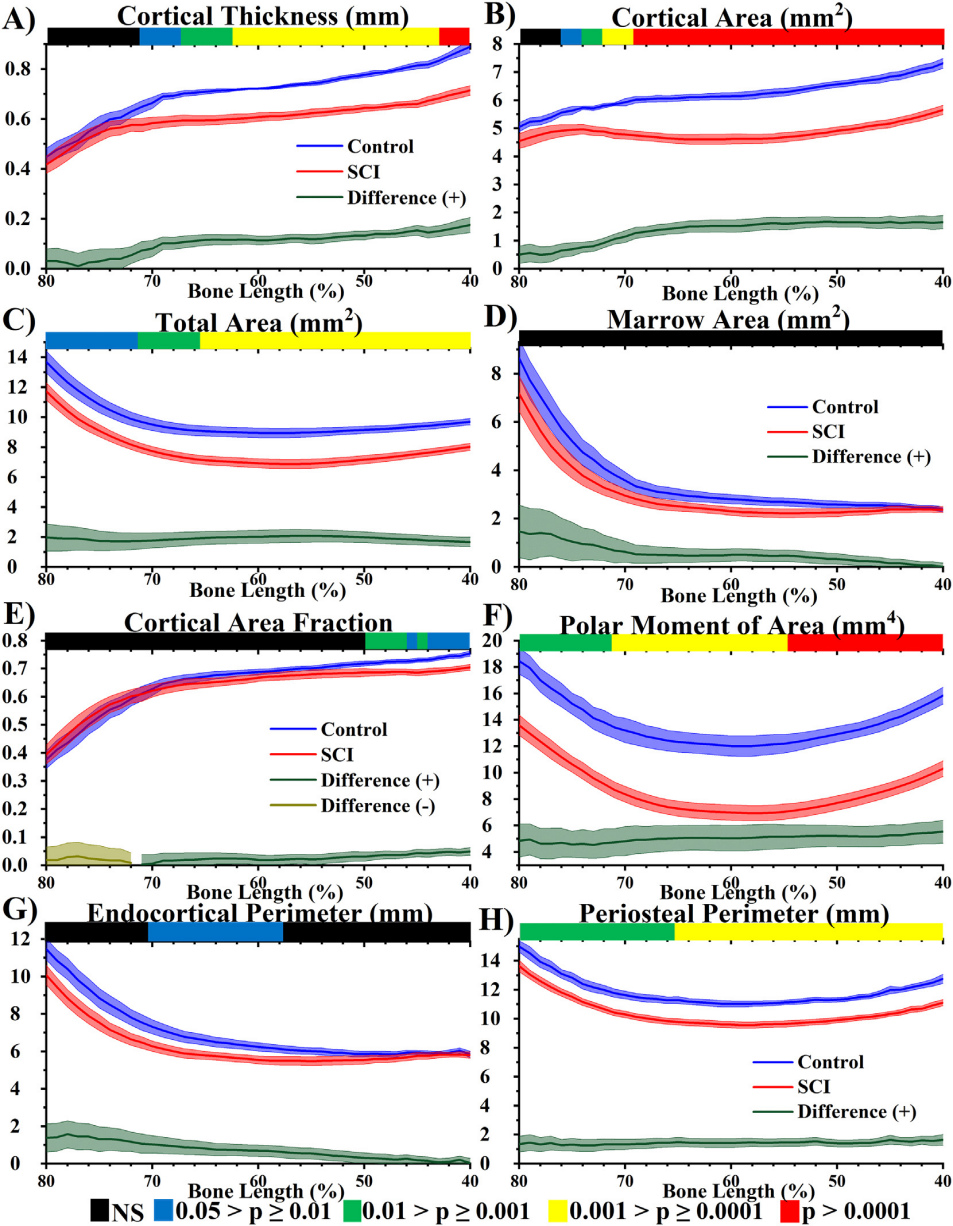
2D morphometric analysis of cortical bone (40 to 80% bone length) at 10 weeks post-surgery, Control in blue and SCI in red (both *n* = 8). The absolute difference distribution (|Control – SCI|) is in green for positive and dark yellow for negative differences. A) Cortical thickness (Ct.Th_2D_), B) Cortical area (Ct.Ar_2D_), C) Total area (Tt.Ar_2D_), D) Marrow area (Ma.Ar_2D_), E) Cortical area fraction (Ct.Ar_2D_/Tt.Ar_2D_), F) Polar moment of area (J_2D_), G) Endocortical perimeter (Ec.Pm_2D_) and H) Periosteal perimeter (Ps.Pm_2D_). Significant levels provided by *p*-value heat maps, where black is non-significant (NS), blue .05 > *p* ≥ .01, green .01 > *p* ≥ .001, yellow .001 > *p* ≥ .0001, red *p* > .0001. (For interpretation of the references to colour in this figure legend, the reader is referred to the web version of this article.)

Cortical bone morphometric parameter distributions were analysed between 40 and 80% bone length from the proximal end of the femur for the Control group (Fig. 5). The trend was for increasing total area and marrow area moving from the mid-shaft towards the distal metaphysis. At 80% bone length Tt.Ar_2D_ and Ma.Ar_2D_ were 49% and 221% greater than at the mid-shaft (60% bone length) (both p < .0001). Ps.Pm_2D_ and Ec.Pm_2D_ distributions confirmed this finding. In contrast, at 80% bone length Ct.Ar_2D_ was 23% lower (*p* < .001) than at the mid-shaft. Regardless of the decreasing cortical area observed moving distally from the midshaft, the changes in the spatial distribution of bone around its centre of gravity (i.e. increasing Tt.Ar_2D_ and Ma.Ar_2D_) led to higher polar moment of area in the more distal regions compared to the midshaft. At 80% bone length J_2D_ was 54% (p < .001) higher than at 60% bone length.

### 3.4 Site-specific cortical bone changes as a result SCI-induced osteoporosis

In comparison to the Control group, the SCI group had significantly lower Ct.Th_2D_ (*p* < .01) and Ct.Ar_2D_ (*p* < .0001) throughout the diaphysis (40–70%), whereas more distally beyond 70% and 76% bone length no significant differences were observed in Ct.Th_2D_ and Ct.Ar_2D_. Tt.Ar_2D_ and Ps.Pm_2D_ were significantly lower (*p* < .05), throughout all cortical regions (40–80%), while no significant difference in Ma.Ar_2D_ and only regional differences in Ec.Pm_2D_ (60–70%) (p < .05) were observed throughout the length. These morphometric parameters contributed to an average 5.0 ± 0.9 mm^4^ reduction in polar moment of area throughout the entire cortical region.

**Fig. 6:**
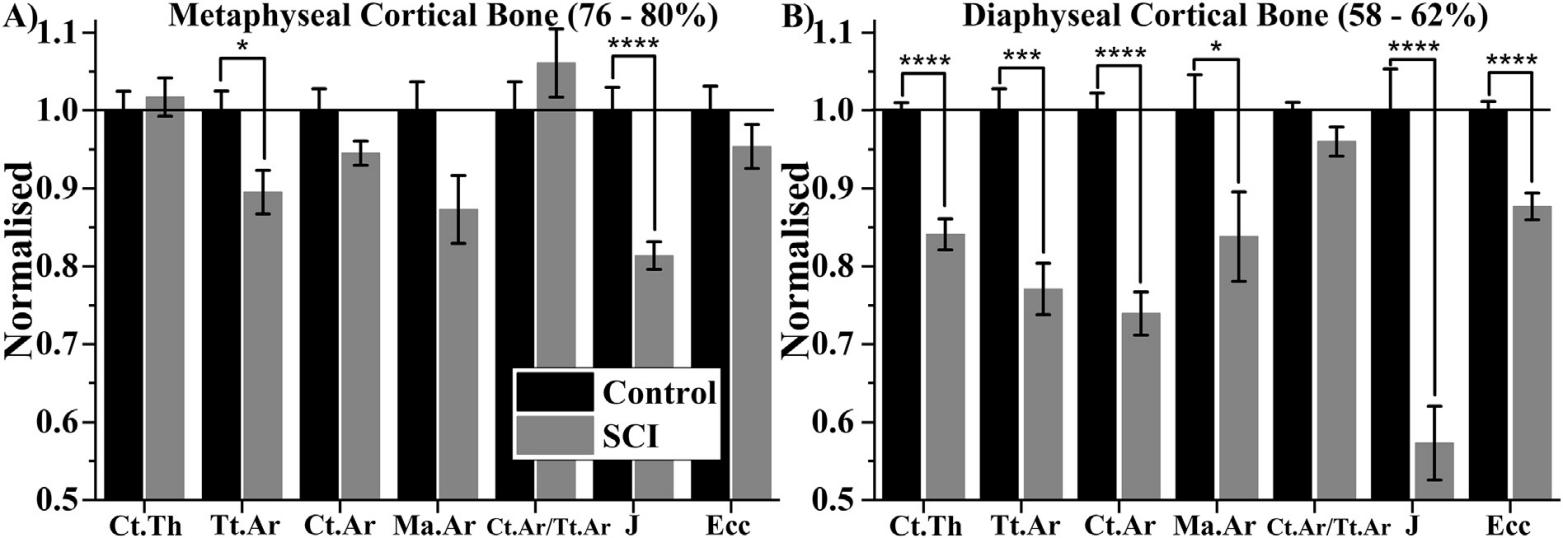
Average 3D morphometric parameters A) metaphyseal and B) diaphyseal cortical bone. All parameters are normalised with respect to the Control group. Ct: Cortical; Th: Thickness; Tt: Total; Ar: Area; Ma: Marrow; J: second polar moment of area; Ecc: Eccentricity. Normalised average ± SE are presented. *,*** and **** indicate p < .05, p < .001 and p < .0001, respectively.

These findings were validated by conventional 3D analysis (Fig. 6). The region identified to represent diaphyseal cortical bone ranged from 58 to 62% bone length. Diaphyseal Ct.Ar was 26% (*p* < .0001) lower in SCI compared Control, characterised by 23% (*p* < .001) and 16% (*p* < .05) lower Tt.Ar and Ma.Ar, respectively. These changes contributed to 16%, 43% and 12% lower Ct.Th, J and Ecc respectively (all p < .0001). In contrast, there was no significant difference in metaphyseal Ct.Ar between Control and SCI. Despite this Tt.Ar was 10% (p < .05) lower, which contributed to a 19% reduction in J (p < .0001).

## 4 Discussion

This study used two approaches to characterise the morphometry of the distal femur in a T9 transection rat model of SCI at 10 weeks post-surgery. Firstly, a global 2D ‘slice-by-slice’ approach was developed and implemented with custom-made MATLAB scripts, where 2D morphometric parameters are expressed as a function of bone length. This allows for the quantification of entire trabecular and cortical structures, maintaining information on the spatial variability and extent of morphometric parameters. This permits the surveying of entire bones, which provides a uniquely detailed description of the bone and its response to SCI-induced osteoporosis. It also allows identification of regions of interest that may prompt further, more computationally expensive 3D analysis. Secondly, this targeted 3D analysis was performed.

The trabecular morphometric distributions in Fig. 2 highlight that the quantity and microarchitecture of this structure in the distal femur can vary considerably over short distances. As trabecular bone extends from the growth plate into the metaphysis and diaphysis it becomes less dense. This decreasing metaphyseal density gradient with distance from the growth plate has previously been quantified in mouse and rat long bones (Gabet et al., 2008; Weber et al., 2002). In this study, the decreasing BA/TA gradient was characterised by a decreasing Tb.N_2D_ gradient throughout, while Tb.Th_2D_ only significantly decreased in the more proximal regions of the metaphyseal trabecular structure (60 to 77% bone length for Control).

There were site-specific effects of SCI-induced osteoporosis at 10 weeks post-surgery on trabecular morphometry throughout the entire distal femur. The metaphyseal BA/TA and Tb.N_2D_ gradients were not significantly altered, although there was significant lowering of the initial maximum height and extent of the distributions. The quantification of significantly lower trabecular extent at all BA/TA measurement points in SCI compared to Control group is new. The value of this measure should be investigated further for its significance and wider applicability through mechanical testing (for example trabecular blunt-end indentation) and finite element analysis. In contrast the epiphyseal compartment was structurally the most resistant to SCI-induced osteoporosis, with less severe reduction in BA/TA.

This varying trabecular landscape demonstrates that appropriate VOI selection for 3D analysis is key to understanding and interpreting the data appropriately. Plotting morphometric functions this way produces a visualisation of the longitudinal spatial variation of bone and can provide confidence for selecting sub-volumes, on which to perform more standard (3D) analyses. Metaphyseal secondary spongiosa sites are the most common trabecular VOIs investigated in rat long bone μCT studies. The VOI is most commonly defined based on a set distance (number of slices) from a reproducible landmark (typically the metaphyseal growth plate) (Bouxsein et al., 2010). To further investigate the effects of SCI-induced osteoporosis on trabecular bone here it was defined based on a percentage bone length scale, thus accounting for variation in bone length and allowing for comparison between similar regions in bones at different phases of skeletal growth. An inherent bias is introduced with percentage-based VOI selection. Longer femurs would have VOIs with a higher total number of slices than shorter femurs (i.e. be oversampled), thus for example, more trabeculae may be present in the VOI of longer that shorter femurs. However, this bias must be judged against the bias introduced by selecting VOIs with a fixed total number of slices, in this case shorter femurs would be oversampled. The appropriate method should be chosen for the research question, in this study the objective was to compare anatomically and biomechanically similar regions.

Results from 3D analysis validated the morphometric distribution findings and further elucidated the structural effects of SCI-induced osteoporosis. The epiphyseal trabecular compartment was structurally more resistant than the metaphyseal compartment, the structural reasoning being that (as seen in the Control) on average the trabeculae in this compartment are significantly thicker and more plate-like, offering less surface area per unit volume on which bone resorption can occur on. Furthermore, the structure's overall connectivity was maintained post-SCI as trabeculae were only thinned, not perforated. This result was in accordance with Lin et al. (2015), who performed the only other rat study of SCI-induced osteoporosis that characterised the effects of SCI at epiphyseal and metaphyseal trabeculae bone sites. In 4-month old male rats that were given T9 contusion injury for a duration of 16 weeks, they observed a much milder deterioration of epiphyseal compared to metaphyseal trabecular bone. The differences between epiphyseal and metaphyseal trabecular compartments observed here were first described at the proximal tibia in 7-month old female rats (Sheng et al., 2007). That study further went on to describe site-specific structural changes in both compartments as a result of ovariectomy-induced osteoporosis (OVX), which are overall in accordance with the site-specific structural changes after SCI described in this study.

The cortical morphometric distributions (Fig. 5) all showed clear variations throughout the cortical length analysed (40 to 80% bone length). Uniform significant lowering of morphometric parameters that indicate activity on the periosteal surface (Tt.Ar_2D_, Ps.Pm_2D_ and to a lesser extent J_2D_) were observed in SCI compared to Control throughout. In contrast, site-specific effects (i.e. differences between the proximal and distal regions of the cortical length) were observed in parameters that indicate activity on the endocortical surface (Ma.Ar_2D_ and Ec.Pm_2D_) (Jepsen et al., 2015). Overall this resulted in site-specific changes in diaphyseal mass accumulation following SCI, with significant lowering of Ct.Th_2D_ and Ct.Ar_2D_ more proximally along the cortical bone, but not distally. These morphological shifts increased femoral resistance to deformation distally in both Control and SCI groups, but resistance to deformation was significantly less in the SCI group as quantified by the lower second polar moment of area. μCT studies conducted into the effects of SCI-induced osteoporosis typically perform cortical morphometric analysis around the midshaft (Jiang et al., 2007; Lin et al., 2015; Liu et al., 2008; Morse et al., 2008), few have analysed the more clinically-relevant (for SCI) metaphysis (Jiang et al., 2006c; Yarrow et al., 2014; Otzel et al., 2019). The results presented here indicate that measurements from the mid-diaphyseal region do not generalise well to the distal end, with 3D analysis on a diaphyseal (58 to 62% bone length) and metaphyseal (76 to 80% bone length) VOI confirming these observations. This is a well-known observation as described in the OVX rat model (Mosekilde et al., 1998).

The trabecular bone results observed here match those of clinical observations of excessive bone loss in trabecular compartments in the adult human SCI population (Coupaud et al., 2009; Eser et al., 2004). In contrast, the cortical bone results do not. Adult humans with SCI experience cortical bone loss through endocortical thinning (marrow expansion). In contrast, periosteal effects were observed here, i.e. a lowering of Tt.Ar, which compares well with the bone changes observed in children with SCI, where bone changes are a combination of bone loss and abnormal bone growth/development (Biggin et al., 2013). This could be explained by the fact that the rats used in this study were relatively young and still undergoing skeletal development.

The value of 2D approaches such as those applied here has been previously shown in the literature. Wu et al. (2015) used a 2D approach to localise differences in the trabecular architecture in the lumbar vertebrae of OVX and sham-operated rats. The region identified as containing the largest microstructural differences between the two groups was further analysed using more computational expensive (3D morphometric and finite element) analysis. While Galea et al. (2015) used a 2D approach to quantify the geometry of cortical bone throughout the entire mouse tibiae from several different experimental groups; including aged, OVX and disuse. They noted site-specific differences between groups that would not have be picked up using the standard procedure of analysis of morphometry at narrow cross-sectional sites.

In conclusion, this is the first ex vivo μCT study to chart the detailed distributions of both trabecular and cortical bone 2D morphometric parameters, and to use it as a basis for VOI selection for further 3D analysis. This technique may be applied to other weight-bearing long bones (e.g. tibia, humerus, radius, ulna), other tomographic imaging modalities (e.g. in vivo μCT, magnetic resonance imaging) and other types of experimental design including the effects of targeted rehabilitation strategies, aging, loading, gene knockout and pharmacological intervention, to identify site-specific structural effects.

## Author roles

Jonathan A. Williams – JAW

James F.C. Windmill – JFCW

K. Elizabeth Tanner – KET

John S. Riddell – JSR

Sylvie Coupaud – SC

Authors' roles: Study design: JAW, JSR, SC. Study conduct: JAW, JSR, SC. Data collection: JAW, JFCW, JSR. Data analysis: JAW, SC. Data interpretation: JAW, KET, SC. Drafting manuscript: JAW, SC. Revising manuscript content: JAW, JFCW, KET, JSR, SC. Approving final manuscript content: JAW, JFCW, KET, JSR, SC. JAW takes responsibility for the integrity of the data analysis.

## Declaration of competing interest

None.

## Appendix A Supplementary data

Supplementary material Image 1
