# Supplementary material for: Global and site-specific analysis of bone in a rat model of spinal cord injury-induced osteoporosis
Source: Bone Rep. 2019 Nov 29;12:100233. doi: 10.1016/j.bonr.2019.100233 (PMC6920718; doi:10.1016/j.bonr.2019.100233)
Supplement: Supplementary file 1 — Supplementary material [file mmc1.docx]

Supplemental Material

**Section 1.** Co-registration protocol

**Section 2.** Segmentation protocol

**Section 3.** 2D morphometric parameters

**Section 4.** Validation of 2D morphometric parameters

**Section 5.**

**Section 1.** Co-registration protocol

Co-registration was used to ensure that the rat femur datasets were all spatially aligned. Femurs are not cylinders, thus when µCT scanned do not sit precisely at the scanner’s isocentre. Reconstructed datasets therefore all have different coordinate systems. Co-registration uses affine transformations to rotate and translate each femur dataset into a standardised position. This procedure was carried out in Dataviewer (Version 1.5.1.9, Kontich, Belgium).

Firstly, a representative μCT dataset was selected from both Control and SCI groups, to act as a reference (standardised position). These datasets were manually aligned such that the z-axis was aligned to the cortical shaft (Figure SM1.1).


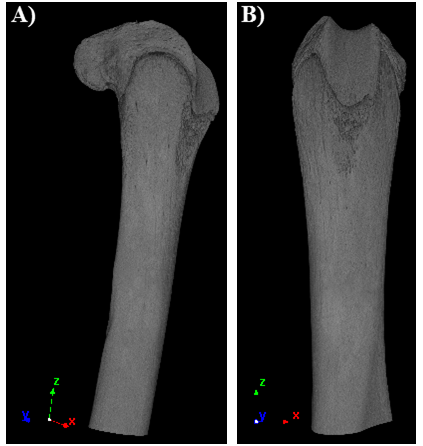


**Figure SM1.1.** Manual alignment of a representative reference distal femur. A) unaligned distal femur. B) Manually aligned so that the femoral shaft is orientated parallel to the image z-axis (in green).

Next, the global shape of the femur was acquired by applying a global threshold at 35% of the maximum grey value to the reconstructed greyscale dataset, followed by a flood-fill operation that limited the region of interest to the bone’s endocortical perimeter. This resulted in a binarised femur dataset without any internal trabecular structure (Figure SM1.2). This operation was applied to both reference dataset and all other (n = 7 for each group) datasets.


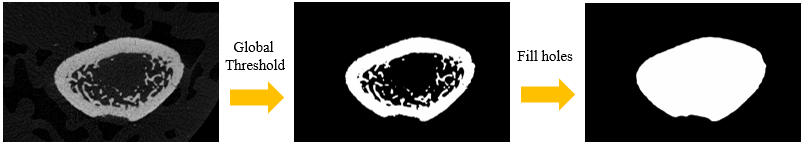


**Figure SM1.2.** Schematic in 2D showing the image processing steps needed to acquire the overall shape of the femur.

Next, the remaining datasets in each group were transformed into the coordinate system of the reference dataset. To speed up this process, these datasets were each individually manually translated and rotated close to that of the reference’s orientation. Automatic matching then could take over. Matching between reference and target dataset was carried out on a small VOI that contains structures, within the datasets. The VOI chosen here was the femoral condyles, due to their characteristic shape. 3D registration was decomposed in to separate 2D tasks, matching (two translations and one rotation) was done iteratively on each of the orthogonal views (Figure SM1.3), once the difference between the two (reference and target) datasets was small in each view the matching stopped. The resulting rigid transformation matrix was then applied to the binarised reference dataset (Figure SM1.3).


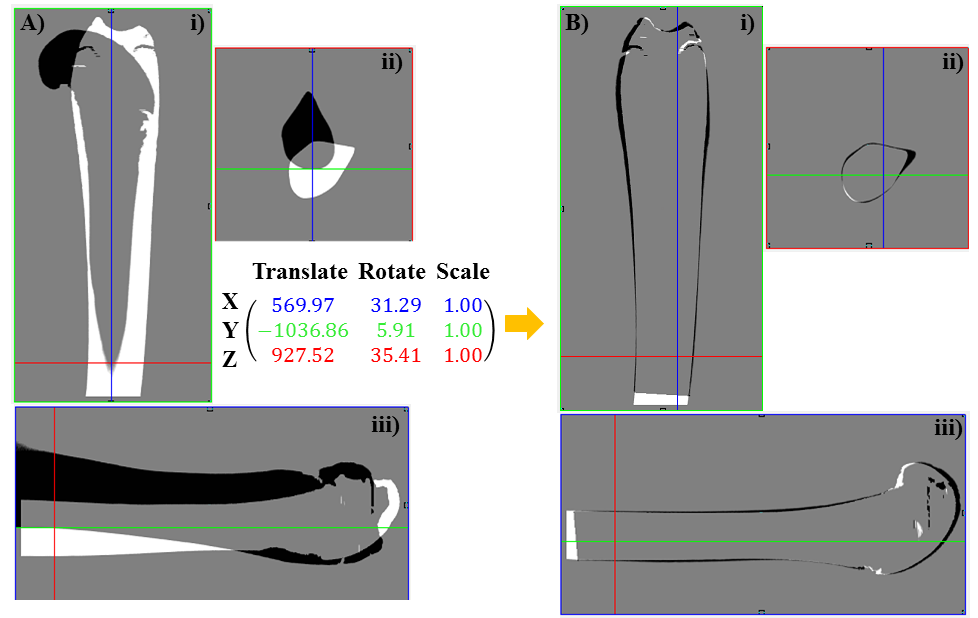


**Figure SM1.3.** Overview of co-registration, shown in i) coronal (X-Z), ii) transverse (X-Y) and iii) sagittal (Z-Y) views. A) Un-aligned datasets, the reference dataset is in white and the target dataset is in black. B) The rigid transformation matrix is applied to transform the target dataset into the reference’s coordinate system.

Finally, this transformation matrix was applied to the original target greyscale dataset (Figure SM1.4). Text rewritten from J.A. Williams, Characterisation of disuse-related osteoporosis in an animal model of spinal cord injury, Doctoral Thesis, University of Strathclyde, Glasgow UK, 2019. Further information on this co-registration methodology is described in MN048 (Method Note, Advanced image co-registration in DataViewer, Bruker)


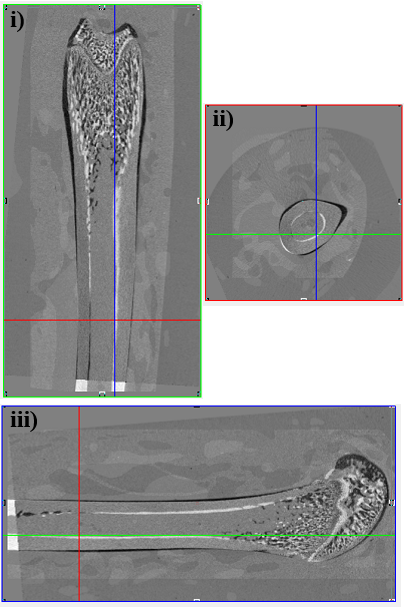


**Figure SM1.4.** Co-registered greyscale distal femurs achieved with the transformation matrix. Shown in i) coronal (X-Z), ii) transverse (X-Y) and iii) sagittal (Z-Y) views.

**Section 2.** Segmentation procedure

An overview of the steps involved in the automated segmentation algorithm implemented in this study is presented below. All thresholds and kernel sizes used were chosen through optimisation for the datasets in question. This algorithm was applied to all reconstructed co-registered greyscale µCT datasets of the rat distal femur and was implemented in CTAn (Version 1.16.10.0, Kontich, Belgium). This overview was optimised from MN008, Automated trabecular and cortical bone selection (Bruker microCT), and taken from J.A. Williams, Characterisation of disuse-related osteoporosis in an animal model of spinal cord injury, Doctoral Thesis, University of Strathclyde, 2019.

#### 3.2.4.1 Trabecular Bone Segmentation

The purpose of this algorithm is to delineate the marrow cavity, while including trabecular bone, but excluding the cortical bone. The algorithm is presented in two parts, each with a specific purpose. Each part is subsequently divided into essential steps.

*Part 1 – Defining the Bone Cross-sectional Area*

Defining the bone was achieved by segmenting the bone tissue from surrounding soft tissue, removing remaining noise, and defining a region of interest (ROI) that encapsulates the bone.

Step 1: Global Thresholding

The µCT datasets acquired are high resolution, and with high contrast between the bone and the surrounding softer tissues (PBS-soaked gauze exterior, marrow fat interior). The partial volume effect is minimal, and a simple global threshold was suitable for segmenting the bone tissue from surrounding softer tissues (Figure SM2.1). The two limits of the threshold range used throughout this study to distinguish bone from non-bone material were 90 – 255 grey levels, corresponding to 35% - 100% of the maximum grey value.


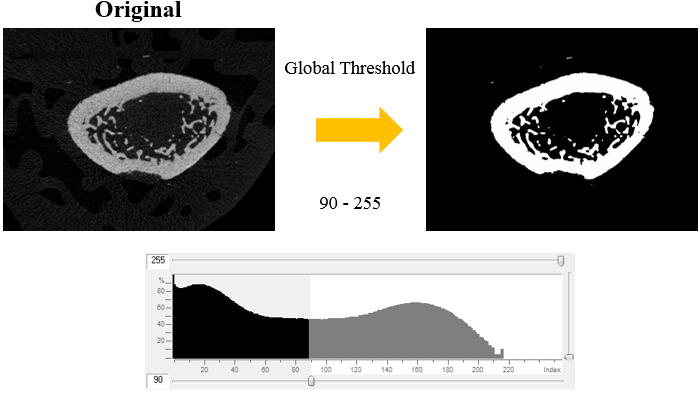


**Figure SM2.1.** Global threshold for segmentation of bone tissue from softer tissues.

Step 2: Noise Removal

Any remaining noise in the binarised dataset was removed with a sweep function which was applied in 3D (to the entire dataset) (Figure SM2.2). This function first found all the connected components (objects) in the binarised dataset, all objects that were not connected to the largest object, i.e. the bone were removed. This binarised segmented bone was the starting point for the “image view” and “internal ROI view” in part 2.


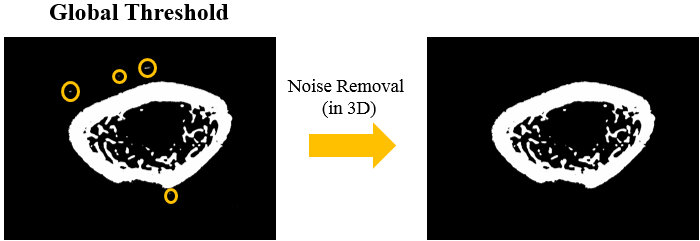


**Figure SM2.2.** Removal of white speckles, by removal of all except the largest object (in 3D).

Step 3: Cortical Bone ROI Selection

A flood-fill function was applied in 2D (slice-by-slice), this functioned to completely fill the marrow cavity and closed pores within the cortical bone. In slices where a pore runs completely through the cortical bone, meaning the marrow cavity is connected to the space outside, e.g. at nutrient foramens, a morphological closing operation with structuring element (kernel) size of 70 pixels was applied. This had the effect of stretching over holes and filling in open pores. Thus, this step limits the ROI to the bone’s periosteal surface (Figure SM2.3) and defines the starting ROI view for part 2 (Figure SM2.4).


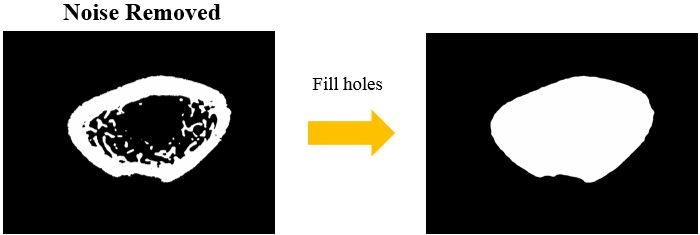


**Figure SM2.3.** Defining the bone’s total cross-sectional area with flood-fill and morphological closing operations.

To help describe the next steps, the three views are now defined. These are the image view, the ROI view and the internal ROI view (Figure SM2.4). The effects each of the following operations have on each of the views will be visualised.


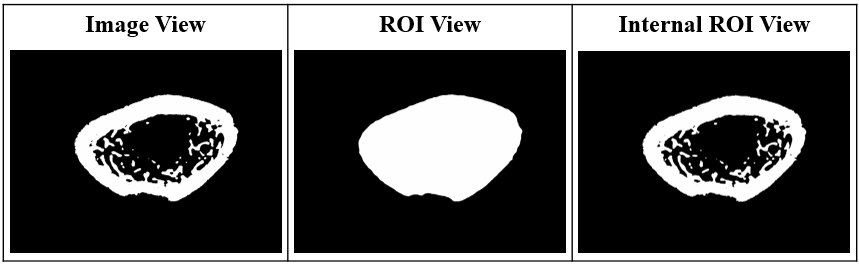


**Figure SM2.4.** The input image view, ROI view and internal ROI view, for part 2 of the segmentation algorithm, the purpose of which is to select the marrow cavity.

*Part 2 – Selection of Marrow Cavity*

Now that the bone cross-sectional area had been defined, the second part of the algorithm selected the marrow cavity.

Step 4: Bitwise NOT Operator

Firstly, the image was inverted with a bitwise operation (Image = **NOT** Image) (Figure SM2.5). White become black and black becomes white.


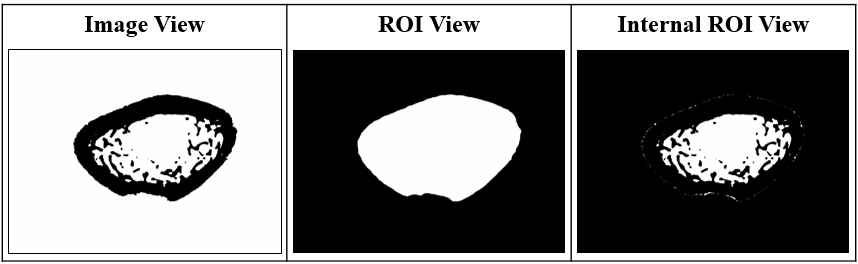


**Figure SM2.5.** Effects of applying an inversion (bitwise NOT operator) to the image.

Step 5: Bitwise AND Operator

The image view shows that the inversion led to the selection of both the marrow cavity and the space exterior to the bone (Figure SM2.5). To limit the marrow cavity selection to just the interior space of the bone a second bitwise operation was applied to the image (Image = Image **AND** ROI) (Figure SM2.6).


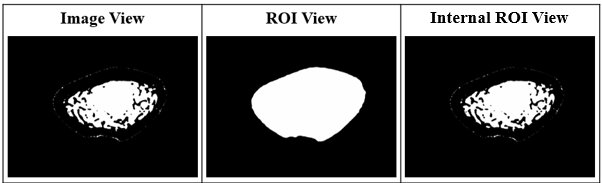


**Figure SM2.6.** Application of the bitwise AND operator to the image view. It compares each pixel in the first operand to that of the corresponding pixel in the second operand. If both pixels are white, then the resultant pixel is white, otherwise it is black.

Step 6: White Speckle Removal

White speckles exist at the periosteal perimeter of the bone in both the image and internal ROI views (Figure SM2.6). This was due to the morphological closing operation that was used in step 3, which stretched over holes and filled in open pores (Figure SM2.3). To remove theses speckles, which are smaller than all marrow cavity objects selected, a morphological opening was applied with a disk-shaped kernel of radius 5 pixels (Figure SM2.7). Opening is defined as an erosion followed by a dilation using the same kernel. This has the effect of removing all objects that cannot be entirely contained by the kernel and forming new boundaries that conform with the disk-like shape of the kernel.


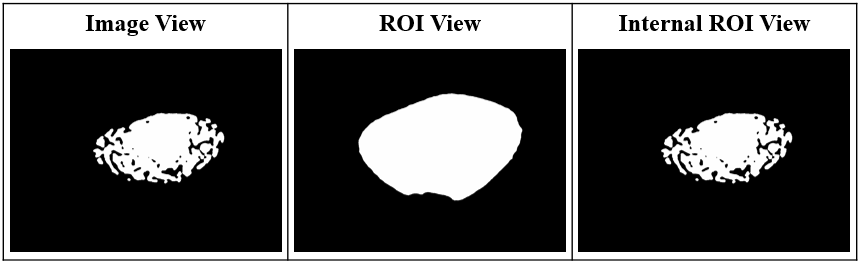


**Figure SM2.7.** Effect of the removal of white speckle with disk-shaped kernel of radius 5 pixels.

Step 7: Inclusion of Trabecular Bone

The empty regions of the marrow cavity have been selected, but the trabecular bone has not (it appears as black gaps within the marrow cavity) (Figure SM2.7). These gaps were filled with a morphological closing operation, with disk-shaped kernel of radius equal to at least half that of the largest gap (Figure SM2.8). A radius of 50 pixels was used here. Closing is defined as a dilation followed by an erosion using the same kernel. Here it had the effect of turning background (black) pixels in to foreground (white) pixels, if the kernel could not completely fit within the background gap.


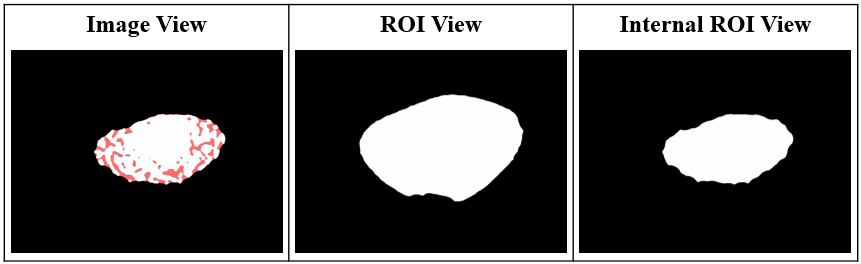


**Figure SM2.8.** Effect of morphological closing with disk-shaped kernel of radius 50 pixels in red, indicating that the gaps have now been filled in.

Step 8: Erosion to Ensure No Cortical Bone Selected

To ensure that the marrow cavity did not contain any cortical bone an erosion with disk-shaped kernel of size 3 pixels was used. This resulted in the bone marrow cavity ROI starting just several pixels from the endocortical perimeter (Figure SM2.9).


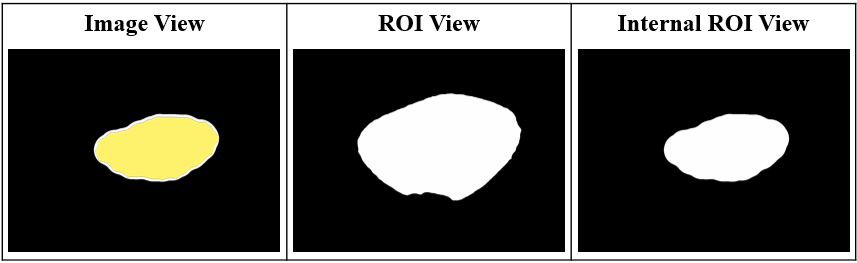


**Figure SM2.9.** Effect of morphological erosion with disk-shaped kernel of radius 3 pixels. Yellow indicates the new image view of the marrow cavity after erosion (not to scale).

Step 9: Reload Original Image into Marrow Cavity ROI

The ROI view was then made a copy of the image view in Figure SM2.9. The original image was then loaded back into this ROI of the marrow cavity thus the trabecular bone had been segmented from the cortical bone (Figure SM2.10).


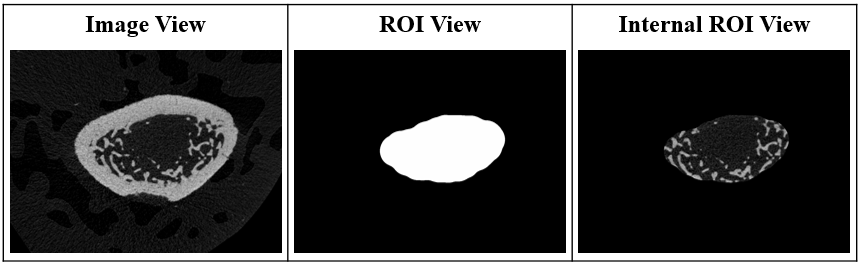


**Figure SM.10.** Effect of reloading original image into marrow cavity ROI.

#### 3.2.4.2 Cortical Bone Segmentation

The segmentation of the cortical bone follows on directly from that of the approach used to segment the trabecular bone, since an ROI has already been defined for the marrow cavity. The procedure is identical up to step 8, thereafter it diverges.

Step 10: Dilation to ensure all cortical bone included

Following on from step 8, a dilation with disk-shaped kernel of size 3 pixels was applied to the ROI (not the image), ensuring all cortical bone was encapsulated within the ROI (Figure SM2.11).


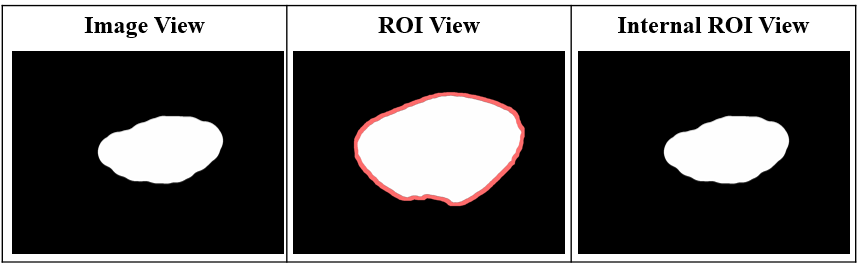


**Figure SM2.11.** Effect of morphological erosion with disk-shaped kernel (not to scale). Red outline representation the increased area.

Step 11: Removal of Marrow Cavity From ROI

The marrow cavity ROI was then subtracted from the dilated ROI, via the bitwise operation ROI = ROI **SUB** Image (Figure SM2.12).


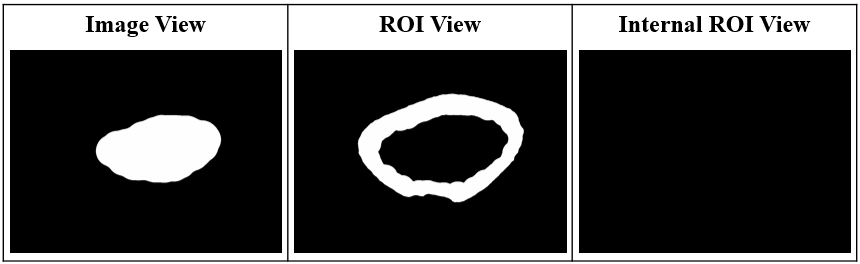


**Figure SM2.12.** Effect of the subtraction of the marrow cavity on the ROI.

Step 12. Reload Original Image into Cortical Bone ROI

The original image was then loaded back into the ROI, as done in Step 9, thus the cortical bone was now segmented from the trabecular bone (Figure SM2.13).


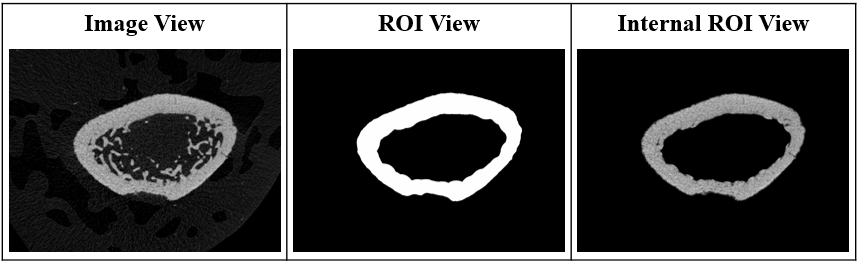


**Figure SM2.13.** Effect of reloading the original image into the ROI.

**Section 3.** 2D morphometric parameters

2D cortical thickness (Ct.Th_2D_) was defined here as the mean distance between the outer (periosteal) and inner (endosteal) bone perimeter for each binarised cross-section. It was determined at four measurement points (0, 90°, 180° and 270°) (Figure SM3.1), then the slice was rotated by 2° about its centroid and Ct.Th_2D_ was measured again at the same four sites. Thus, gull coverage was achieved with 44 rotations. Quantisation error was minimised by duplicating each original unrotated cross-section 44 times, so that each cross-section was rotated only once, but through each of the 45 different angles.


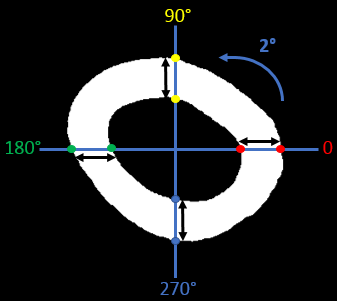


**Figure SM3.1.** Representative cortical cross-section, depicting the cortical thickness calculation. It was determined as the difference between each set of points at the four orientations depicted, a rotation of 2° was then applied and four more measurements were taken, further 2° rotations were applied until complete coverage was achieved.

Ct.Ar_2D_, Tt.Ar_2D_ and Ma.Ar_2D_ were determined by calculating the total number of pixels representing each area in the cross-section (Figure SM3.2). To acquire Figure SM3.2A, a filling procedure was applied to the cross-section to stretch over any pores (holes under a certain radius, i.e. 15 pixels), so the reported Ct.Ar_2D_ ignores pores. To acquire Figure SM3.2B, the same filling procedure was applied, but with a much larger radius (e.g. 200 pixels). To acquire Figure SM3.2C, the XOR Boolean operator was applied to A) and B).


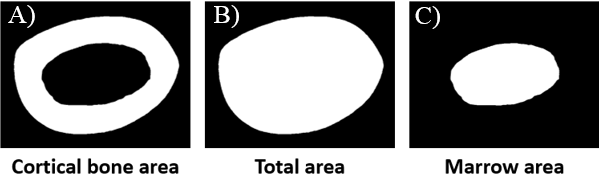


**Figure SM3.2.** A) Representative cortical midshaft cross-section (cortical bone area), B) filling procedure applied to depict total area enclosed by the periosteum, C) XOR Boolean operator applied to show the marrow area.

Counting border pixels is not an effective way to determine perimeters, it would underestimate the boundary length. This is because it considerably reduces the information pertaining to the contour of the original object. This type of perimeter measure would consider all border pixels as equal distances apart (4-connectivity). A more realistic determination of perimeter would be achieved using 8-connectivity, which counts the diagonal lengths between pixels. These lengths are $\sqrt{2}$ times longer than the horizontal and vertical lengths and account for the exact pixel centre to pixel centre lengths of our segmented binarised representations of the original bone. This approximates the boundary to straight lines at orientations 0, 45° and 90°, which is not a true representation of the bone. To reduce this orientation dependency different weightings have been proposed for diagonal and vertical (and horizontal) lengths, and a count of the number of times the orientation changes (Proffitt and Rosen, 1979; Vossepoel and Smeulders, 1982). This is how MATLAB ‘regionprops’ perimeter function determines the perimeter. Ps.Pm is determined on Figure SM3.2B and the Ec.Pm on Figure SM3.2C.

The polar moment of area was calculated in 2D (like the perimeter). The second moments of area were calculated without any assumption of the underlying geometry. Consider Figure SM3.3, $I_{yy}$ symbolises the second moment of area relative to Y-axis, for digitised cross-sections it can be computed as the following summation over all bone pixels.

$$I_{yy}= \sum_{i=1}^{N} \left( Pixel Area \times\left( x_{i}-x_{c} \right)^{2}+Pixel MOA \right)$$

Where, $Pixel Area =Pixel Width \times Pixel Height$, $x_{c}$ is the X-axis coordinate of the bone cross-section centroid, the quantity $x_{i}-x_{c}$ represents the distance from the Y-axis to the i^th^ pixel, $Pixel Moment of Area={\left( Pixel Width \right)^{4}}/{12}$. $I_{xx}$ and $I_{xy}$, the product moments of area, were calculated in a similar fashion.

(S3.1)

The maximum and minimum second moments of area were derived with respects to the relative second moments of area, for example:

(S3.2)

$$I_{max}= \frac{I_{xx}+I_{yy}}{2}+ \sqrt{\left( \frac{I_{xx}-I_{yy}}{2} \right)^{2}+ I_{xy}^{2}}$$

The second polar moment of area follows

(S3.3)

$$J= I_{max}+I_{min}$$

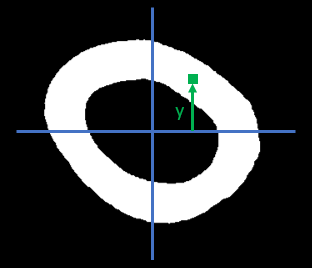


**Figure SM3.3.** Representative cortical cross-section, depicting the calculation of moments of area. Each pixel is a known distance from the centroid, thus allowing moments of area to be determined.

In 2D analysis trabecular BA/TA was defined as the ratio of the total number of pixels representing trabecular bone to the total number of medullary cavity pixels, as depicted in Figure SM3.4. It represents the fraction of each medullary cross-section that is occupied by mineral bone tissue.


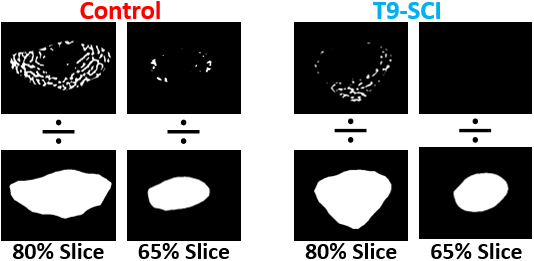


B)

A)

**Figure SM3.4.** Schematic of trabecular bone area fraction (BA/TA). For representative A) 10-week control and B) 10-week SCI femurs at 80% and 65% bone length from the proximal end.

Tb.Th_2D_ and Tb.Sp_2D_ were determined with an analogous method to that of the maximum sphere fitting method used to determine 3D thickness and separation (Hildebrand and Rüegsegger, 1997). That is fitting maximum circles to all points within the bone structure of each µCT slice, these are circles that include the point and entirely fit within the structure. Tb.Th_2D_ was then the average diameter of all these circles. Tb.Sp_2D_ was determined with the same technique, just applied to the inverse of the structure. Tb.N_2D_ was determined from the 2D thickness and separation measurements using the following equation:

$${Tb.N}_{2D}= \frac{1}{({Tb.Th}_{2D}+{Tb.Sp}_{2D})}$$

This section was taken from J.A. Williams, Characterisation of disuse-related osteoporosis in an animal model of spinal cord injury, Doctoral Thesis, University of Strathclyde, 2019.

**Section 4.** Validation of 2D morphometric parameters

The custom-made MATLAB scripts calculate 2D morphometric parameters for each slice in a VOI and plots them versus percentage bone length, while CTAn calculates the 2D morphometric parameters for each slice in a VOI and averages the values, reporting the mean. To validate the custom MATLAB scripts, a comparison was made with CTAn software. The following equation was used to do this:

(C.1)

$${Average}_{2D Parameter}=\frac{\sum_{i=1}^{N} {2D Parameter}_{i}}{N}$$

where, $N$ is the number of slices making up the VOI, ${2D Parameter}_{i}$ is the 2D morphometric value at the $i^{th}$ slice. This equation therefore converts the 2D morphometric distributions into means, allowing comparison with CTAn. For 10-week control group trabecular (81 – 85% bone length) and cortical (62 – 58% bone length) VOIs, both MATLAB-determined and CTAn-determined 2D morphometric parameters were compared (Table SM4.1).

**Table SM4.1.** Comparison of MATLAB- and CTAn-determined morphometric parameters for the 10-week control group. Data presented as mean ± SE.

| 2D Parameter | MATLAB-determined | CTAn-determined |
| --- | --- | --- |
| BA/TA (%) | 28.2 ± 2.3 | 28.2 ± 2.3 |
| **Tb.Th_2D_ (mm)** | **0.06 ± 0.01** | **0.07 ± 0.01** |
| **Tb.Sp_2D_ (mm)** | **0.15 ± 0.02** | **0.26 ± 0.03** |
| **Tb.N_2D_ (mm^-1^)** | **3.9 ± 0.3** | **4.2 ± 0.3** |
| **Ct.Th_2D_ (mm)** | **0.65 ± 0.03** | **0.76 ± 0.03** |
| Ct.Ar_2D_ (mm^2^) | 6.1 ± 0.1 | 6.1 ± 0.1 |
| Tt.Ar_2D_ (mm^2^) | 9.0 ± 0.2 | 9.0 ± 0.2 |
| Ma.Ar_2D_ (mm^2^) | 2.9 ± 0.2 | 2.9 ± 0.2 |
| Ps.Pm (mm) | 11.6 ± 0.4 | 11.6 ± 0.4 |
| Ec.Pm (mm) | 18.3 ± 0.5 | 18.3 ± 0.5 |
| J (mm^4^) | 12.1 ± 0.7 | 12.1 ± 0.7 |

**Bold text** shows parameters where differences were seen between the two approaches.

For trabecular BA/TA and the cortical parameters Ct.Ar_2D_, Tt.Ar_2D_, Ma.Ar_2D_, Ps.Pm, Ec.Pm, and J the two approaches returned the exact same values (Table C.1). While for Tb.Th_2D_, Tb.Sp_2D_, Tb.N_2D_ and Ct.Th_2D_ the values determined by the two approaches were different but not significantly so. The difference existed because the methods used to calculate the parameters were different (but both valid). These parameters were calculated assuming a plate model in CTAn (Parfitt et al., 1987), while in the MATLAB scripts they are calculated as geometry independent measures.

## C.2 Comparison of Methods

To assess how well the gold standard 2D average trabecular BA/TA (mean ± standard error) describes the trabecular bone within VOIs (10% bone length for metaphyseal trabecular bone, 6% bone length for epiphyseal trabecular bone), it was compared with the 2D average trabecular BA/TA distribution, over the same VOIs for both 10-week Control and SCI groups (Table SM4.2). The gold standard 2D average trabecular BA/TA did not adequately describe the variation in trabecular bone quantity within trabecular compartments. For example, in the 80 - 90% VOI the recorded gold standard average BA/TA was 25% lower (p < 0.001) than at the distal-most end and 74% higher (p < 0.0001) than at the proximal-most end of the VOI, for control. A similar but greater discrepancy was observed in the SCI VOI, where BA/TA was 43% lower (p < 0.001) at the distal-most end and 294% higher (p < 0.0001) at the proximal-most end of the VOI, compared to the gold standard average value. Similar trends were observed for other trabecular morphometric parameters (data not shown).

**Table SM4.2.** Comparison of the 2D slice-by-slice approach with the integrated gold standard approach for trabecular bone area fraction (BA/TA), for both 10-week control (CTL) and SCI groups. Data presented as mean ± SE.


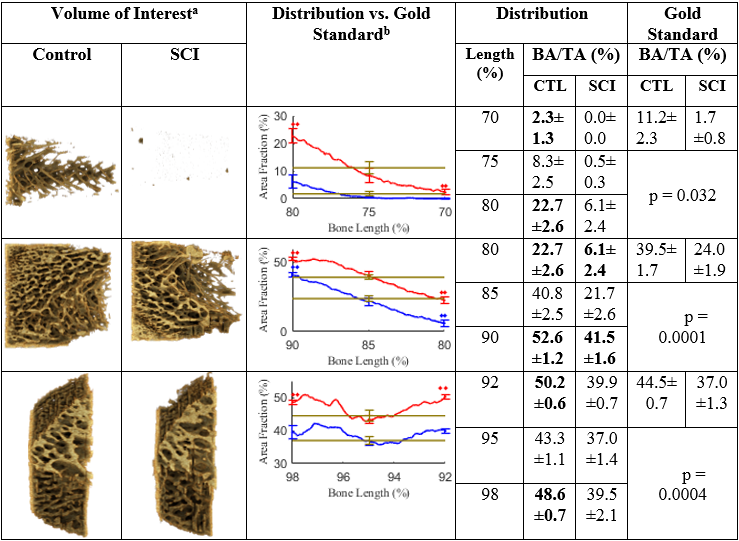


^a^ 3D visualisations of representative µCT 10% bone length metaphyseal trabecular VOIs, and 6% epiphyseal trabecular bone VOIs for both control and SCI at 10-weeks post-surgery.

^b^ Graphical comparison of the average trabecular BA/TA distribution against the gold standard integrated value. control in red, SCI in blue and corresponding gold standards in gold.

**Bold text** refers to a significant difference between slice-by-slice approach and gold standard (p < 0.05).

To assess how well the cortical bone is described by the gold standard 2D average cortical morphometric parameters within VOIs (each 10% bone length), the average Tt.Ar was compared with the 2D average Tt.Ar distribution over the same VOI for both 10-week control and SCI groups (Table C.3). For the diaphysis (70 – 40% bone length) the variability of Tt.Ar was adequately described by the gold standard with the 10% bone length VOIs. It was only more distally (70 - 80%) where the Tt.Ar varies at a faster rate per unit length, that the gold standard does not perform adequately. For control the gold standard average 2D Tt.Ar was 26% lower (p < 0.001) than at the most-distal end and 15% higher (p < 0.01) than at the most-proximal end of the 10% bone length VOI. Whilst for (70 - 80%) SCI VOI the gold standard Tt.Ar was 26% lower (p < 0.001) than at the most-distal end and 10% lower (p < 0.01) than at the most-proximal end of the 10% bone length VOI. All other cortical morphometric parameters displayed a similar trend when compared with the gold standard, i.e. statistical differences were detected in the most distal (70 - 80%) VOI (data not shown).

**Table C.3.** Comparison of the 2D slice-by-slice approach with the integrated gold standard approach for total cortical bone cross-sectional area inside the periosteal envelope (Tt.Ar), for both 10-week control and SCI groups. Data presented as mean ± SE.


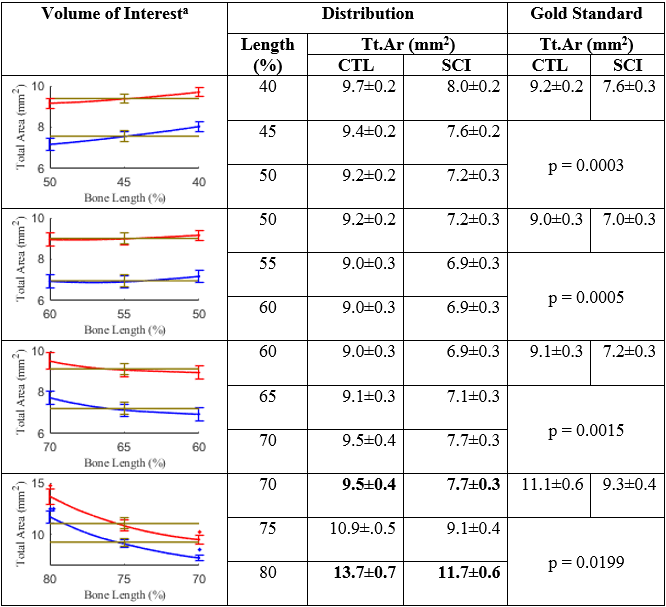


^a^ Graphical comparison of the total area distribution against the gold standard integrated value. Control in red, SCI in blue and corresponding gold standards in gold.

**Bold text** refers to a significant difference (p < 0.05) between slice-by-slice approach and gold standard.

This section was taken from J.A. Williams, Characterisation of disuse-related osteoporosis in an animal model of spinal cord injury, Doctoral Thesis, University of Strathclyde, 2019.

**Section 5**


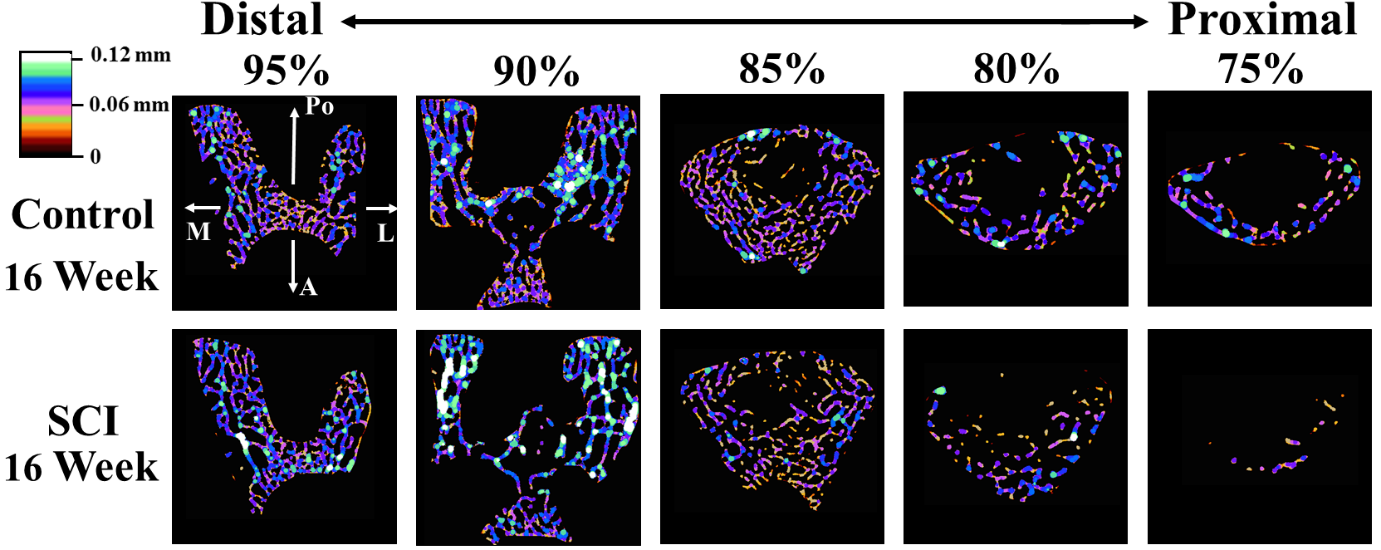


**Figure SM5.1.** Cross-sectional segmented trabecular bone slices with Tb.Th_2D_ mapped by colour, for representative 10-week post-surgery Control and SCI groups at 5% bone length intervals from 95% - 75% bone length from the proximal end of the rat femur. Po: posterior, A: anterior, M: medial and L: lateral.

# Reference

Hildebrand, T., Rüegsegger, P., 1997. A new method for the model-independent assessment of thickness in three-dimensional images. J. Microsc. 185, 67–75. https://doi.org/10.1046/j.1365-2818.1997.1340694.x

Parfitt, A.M., Drezner, M.K., Glorieux, F.H., Kanis, J.A., Malluche, H., Meunier, P.J., Ott, S.M., Recker, R.R., 1987. Bone histomorphometry: Standardization of nomenclature, symbols, and units. Report of the ASBMR histomorphometry nomenclature committee. J. Bone Miner. Res. 2, 595–610. https://doi.org/10.1002/jbmr.5650020617

Proffitt, D., Rosen, D., 1979. Metrication errors and coding efficiency of chain-encoding schemes for the representation of lines and edges. Comput. Graph. Image Process. 10, 318–332. https://doi.org/10.1016/S0146-664X(79)80041-6

Vossepoel, A.M., Smeulders, A.W.M., 1982. Vector code probability and metrication error in the representation of straight lines of finite length. Comput. Graph. Image Process. 20, 347–364. https://doi.org/10.1016/0146-664X(82)90057-0
